# Preliminary Biochemical Description of Brain Oxidative Stress Status in Irritable Bowel Syndrome Contention-Stress Rat Model

**DOI:** 10.3390/medicina55120776

**Published:** 2019-12-06

**Authors:** Ioana-Miruna Balmus, Radu Lefter, Alin Ciobica, Sabina Cojocaru, Samson Guenne, Daniel Timofte, Carol Stanciu, Anca Trifan, Luminita Hritcu

**Affiliations:** 1 Interdisciplinary Research Department–Field Science, “Alexandru Ioan Cuza” University of Iasi, Carol I Avenue, 20A, 700490 Iasi, Romania; balmus.ioanamiruna@yahoo.com; 2 Department of Research, Faculty of Biology, “Alexandru Ioan Cuza” University of Iasi, Carol I Avenue, 20A, 700490 Iasi, Romania; radu_lefter@yahoo.com; 3 Romanian Academy, Iasi Branch, Nr. 8, Carol I Avenue, no. 8, 700490 Iasi, Romania; stanciucarol@yahoo.com; 4 Department of Biology, Faculty of Biology, “Alexandru Ioan Cuza” University of Iasi, Carol I Avenue, 20A, 700490 Iasi, Romania; sabina.cojocaru@uaic.ro; 5 Department of Biochemistry and Microbiology, University Ouaga I Pr Joseph KI-ZERBO, Dagnöen Nord, Ouagadougou BP 7021, Burkina Faso; guesams@gmail.com; 6 Faculty of Medicine, “Gr. T. Popa” University of Medicine and Pharmacy, 6th University Street, 700490 Iasi, Romania; 7 Department of Gastroenterology, Faculty of Medicine, “Gr. T. Popa” University of Medicine and Pharmacy, 6th University Street, 700490 Iasi, Romania; ancatrifan@yahoo.com; 8 Department of Clinics, Faculty of Veterinary Medicine, University of Agricultural Sciences and Veterinary Medicine “Ion Ionescu de la Brad” of Iasi, 3rd Mihail Sadoveanu Alley, 700490 Iasi, Romania; lumidih@yahoo.com

**Keywords:** contention-stress, rat model, irritable bowel syndrome, oxidative stress, superoxide dismutase, glutathione peroxidase, malondialdehyde, lipid peroxidation, nortriptyline

## Abstract

*Background and objectives:* Oxidative stress and inflammation have been implicated in the etiology of irritable bowel syndrome (IBS), a common gastrointestinal functional disease. This study aimed to further characterize the contention-stress rat model by exploring a possible correlation between oxidative stress markers measured in brain tissues with behavioral components of the aforementioned model. Thus, it is hereby proposed a possible IBS animal model relevant to pharmacological and complementary medicine studies. *Materials and Methods:* Wild-type male Wistar rats (*n* = 5/group) were chronically exposed to 6-hour/day contention, consisting of isolating the animals in small, vital space-granting plastic devices, for seven consecutive days. Following contention exposure, temporal lobes were extracted and subjected to biochemical analyses to assess oxidative stress-status parameters. *Results:* Our results show increased brain oxidative stress in contention-stress rat model: decreased superoxide dismutase and glutathione peroxidase activities and increased malondialdehyde production in the IBS group, as compared to the control group. Furthermore, the biochemical ratios which are used to evaluate the effectiveness of an antioxidant system on oxidative stress could be described in this model. *Conclusions:* The correlations between the behavioral patterns and biochemical oxidative stress features could suggest that this may be a complex model, which can successfully mimic IBS symptomatology further providing evidence of a strong connection between the digestive system, enteric nervous system, and the central nervous system.

## 1. Introduction

Presently, the need for valid animal models for burdensome diseases that lack effective treatment is among the most pressing in research. A viable animal model must reflect disease background to a sufficient degree and feature minimum variability in order to ensure repeatable and reproducible results [1]. Furthermore, in complementary and alternative studies, a valid animal model must be as thoroughly described as possible in order to allow for the screening of positive effects as well as side effects [2]. In this manner, a variety of biomedical research animal models mimicking the clinical features of disorders have been established.

Irritable bowel syndrome (IBS) is a common functional gastrointestinal disease exhibited by 5–10% of the populations in most European countries, the United States of America, and China [3]. Although this syndrome is characterized by distressful symptomatology: abdominal pain, abdominal discomfort, constipation/diarrhea or alternation in stool consistency, and the presence of mucus in stool are the main IBS diagnostic criteria according to Rome IV [4]. However, no report has associated IBS with intestinal damage or carcinogenic effects [5]. The spectrum of IBS etiologies encompasses digestive, enteric, and central nervous system risk factors; hitherto, these were partly accounted as nutritional, hormonal, neurological, psychological, and idiopathic factors [6,7].

Stress is reportedly one of the most important risk factors underlying IBS occurrence [8]. The establishment of several important IBS animal models has thus been based on exposure to stressful conditions or stimuli in order to obtain gastrointestinal symptomatology [9]; such models evidence a clear link between the central and enteric nervous systems [10]. However, since IBS might be more complex than was previously thought, with rich neurological and psychological components, a thorough behavioral description is provided in our previously published report on the contention-stress rat model [11].

Several previous studies implicate oxidative stress and inflammatory features in the etiology of IBS [12,13,14,15]. However, the oxidative stress role in IBS was not fully understood due to the many implications that the other components of IBS could have on the yet nonspecific oxidative changes. For example, it was also demonstrated that gut microbiome suppression could lead to important changes in oxidative status [16,17].

There is no curative or etiological treatment for IBS. The treatment of IBS is based on a supportive relationship with the patient, establishing realistic goals, using both nonpharmacological as well as pharmacological strategies. The pharmacological armamentarium includes bulking agents, antidiarrheal agents, probiotics, antibiotics, antispasmodic, and antidepressants which are used based on individual patient symptomatology. Antidepressants are traditionally used in IBS for ameliorating the pain. These agents alter pain perception by a central modulation of visceral afferents and have the advantage of treating also the associated psychologic symptoms.

This study aimed to explore a possible correlation between the expression of oxidative stress markers in brain tissues with the behavior of the contention-stress rat model. We herein describe the correlations between all three components essential to a valid model gastrointestinal, psychiatric, and biochemical in an IBS animal model. Moreover, we tested the administration of NOR to verify the following hypothesis: changes in oxidative stress status correlate with behavioral patterns in the contention-stressed animals.

## 2. Methods

### 2.1. Animals and Procedures

All the experiments and procedures were performed in accordance with European Regulations and national laws regarding the use of animal models in biomedical research and efforts made to reduce the number of animals that were used. Approval for this study was received from the local ethics committee (USAMV Iasi no. 385/04.04.2019).

Fifteen wild-type male Wistar rats (*Rattus norvegicus*) were randomly selected and assigned to one of the three groups: control (C), IBS model (IBS), or IBS + nortriptyline (NOR). The IBS model was based on a previously described protocol [18], which relies on gastrointestinal disturbance inducing long-lasting contention stress; rats in both the IBS and NOR groups underwent this procedure. NOR was orally administered at a dose of 10 mg/kgBW to NOR group animals to test the reversibility of modulated IBS symptoms by antidepressant agent administration, while the other two groups received saline solution in equivalent volumes [19,20].

For stress exposure, the animals were placed in plastic movement-preventing containers, which granted vital space and limited risk of asphyxiation, in 6-hour immobilization periods for seven consecutive days as previously described by Mozaffari et al. [18].

Following stress induction, the animals underwent previously described behavioral tests: Y maze test (short-term memory performance assessment), elevated plus maze test (anxiety-like behavior assessment), forced swim test (anxiety/depressive-like behaviors assessment), open field test (anxiety-like and disinhibited behaviors assessment), and three-chambers social test (social behavior assessment) [11]. In this way, the results of the behavioral tests refer to: swimming time (SWT) which is a behavior observed in forced swimming test indicating calm, detached behavior, extensively considered as the normal behavior in which the animal explores the possibilities to escape (by difference to struggle that indicates anxiety-like behavior or floating, which indicates depressive-like behavior); number of crossings (CRO), which is a behavioral parameter observed in open field test indicating the general mobility of the animal, its decrease being considered as an indicator for anxious/depressive behavior; rearing behavior (REAR) as observed in open field test stands for the exploration tendency, its increase being considered as an indicator for disinhibited behavior; stretching behavior (STR) as also observed in open field test, which could be considered an anxiety-like behavior due to the uncertainty exhibited in their exploration; grooming behavior (GRO) is generally associated with stress-coping behavior, with its increase indicating the presence of anxiogenic stimuli; empty room time (ERT), which is a behavioral parameter observed in trichamber social test indicating the social isolation tendency of the animal (in the first phase of the test, the animal must choose between social interaction in one room and social isolation in another room), stranger room time (SRT) which is a behavioral parameter also observed in trichamber social test indicating the novelty in social preference (in the second phase of the test, the animal must choose between social interaction with a known animal and social interaction with a new animal).

The animals were then intraperitoneally anesthetized with ketamine (100 mg/kgBW)/xylazine (10 mg/kgBW) according to standard procedures, their temporal lobes were processed using the buffered tissue-extraction method, and the tissue samples were kept at −22 °C until biochemical analysis [21].

### 2.2. Biochemical Analysis

Superoxide dismutase (SOD), glutathione peroxidase (GPx) activities, malondialdehyde (MDA) levels, and total protein levels (PROT) were assessed using previously described methods [22].

SOD enzymatic activity was measured by the indirect method of competitive inhibition with a commercial assay (SOD Assay Kit, Sigma-Aldrich Inc., Saint Louis, MO, USA). Based on the Dojindo’s tetrazolium salt (WST-1) reaction with superoxide anion, the method measures the O_2_ reduction rate inhibited by SOD. In this way, 20 µL buffered temporal lobe brain extract and 20 µL enzyme working solution were added to 200 µL WST working solution and incubated at 37 °C for 20 min. Following incubation, the inhibition activity determined by colorimetric evaluation of soluble formazan dye formation has an endpoint at 450 nm (Analytik Jena Specord 200, Analytik Jena, Jena, Germany). The measurements were performed according to the manufacturer’s instructions.

Similarly, GPx activity was determined by an indirect method of substrate consumption dynamic observation in which the rate of NADPH consumption during the considered time unit could be used as an indicator for GPx activity. GPx Cellular Activity Assay Kit (Sigma, Saint Louis, MO, USA) was used, and all the manufacturer’s instructions were followed using a Beckman Coulter DU-700 series (Beckman Coulter, Mississauga, Canada) spectrophotometric system. Thus, 50 µL buffered temporal lobe extract and 50 µL NADPH assay reagent were added to 890 µL assay buffer. Afterward, the reaction was started by the addition of 10 µL *tert* butyl-hydroperoxide 30 mM and the decrease in absorbance at 340 nm was followed by a kinetic program with an initial delay of 10 s (number of reading: 6, reading interval: 10 s) (Analytik Jena Specord 200, Analytik Jena, Jena, Germany). GPx activity was expressed as GPx enzyme units/mL (U/mL).

Malondialdehyde (MDA) levels were determined by thiobarbituric acid reacting substances assay. 200 μL of buffered temporal lobe extract was added and mixed with 1 mL of 50% trichloroacetic acid, 0.9 mL of Tris–HCl (pH 7.4), and 1 mL of 0.73% thiobarbituric acid. Following the incubation period (100 °C for 20 min), the reaction samples were centrifuged at 3000 rpm for 10 min and supernatant read at 532 nm, in a UV–VIS spectrophotometrical system (Beckman Coulter, Mississauga, Canada).

Total soluble protein levels were determined using typical Bradford assay method in which 200 µL buffered brain extract is added to 600 µL Bradford reagent and its absorbance was read within 30 min at 595 nm. The results were calculated against a bovine serum albumin etalon curve and expressed as mg proteins/mL.

In addition, two biochemical parameters providing data on the intensity and ratios of oxidative processes were calculated using the available biochemical markers: lipid peroxidation/antioxidant activity (MDA per GPx activity ratio) and hydrogen-peroxide production/hydrolysis ratios (SOD per GPx activities ratio) [23,24].

### 2.3. Statistical Analysis

All numerical results were expressed as mean ± standard error of the mean (SEM) after one-way and two-way analysis of variance analysis. For intergroup differences significance, Bonferroni correction was used. Outliers analysis was carried out using maximum normalized residual Grubbs test. Correlations between biochemical parameters (SOD, GPx, MDA, and total soluble proteins) and behavioral parameters (as described in our previous work [11]: the Y Test, Forced Swim Test, Elevated Plus Maze Test, Open Field Test, and Trichamber Social Test) were calculated using Pearson’s correlation in a specialized statistical analysis software (Minitab 17, Coventry, UNITED KINGDOM) in the all-groups comparison method and considered relevant for linear regression analysis. Curvilinear correlations were carried out using specialized statistical analysis software. 

## 3. Results

The IBS group exhibited a significant decrease in SOD enzyme activity in brain tissue relative to the control group, while nortriptyline administration in rats subjected to contention partially reversed the stress-induced change in SOD activity [F (2, 14) = 10.17; *p* = 0.0026] (as shown in Table 1). Similar results were observed with respect to the GPx enzyme activity in brain tissue: chronic contention-stress-induced decrease in antioxidant activity of this enzyme was partially reversed with nortriptyline administration [F (2, 14) = 7.29; *p* = 0.008]. We also observed a visible increase in the intensity of lipid peroxidation processes in the brain tissue of the IBS group relative to that in the control group [F (2, 14) = 8.12; *p* = 0.0058]. Brain tissue total soluble protein levels followed a similar pattern: a significant stress-induced increase in the IBS group was partially reversed by nortriptyline administration [F (2, 14) = 5.77; *p* = 0.018] (as shown in Table 1).

Biochemical ratios were calculated from the available measured parameters; we observed no significant differences in brain tissue SOD/GPx between the study groups. However, we observed that the IBS group exhibited an increased LPO/GPx relative to the control group, whereas nortriptyline administration normalized this ratio [F (2, 14) = 8.48; *p* = 0.005] (as shown in Table 1).

**Figure 1:**
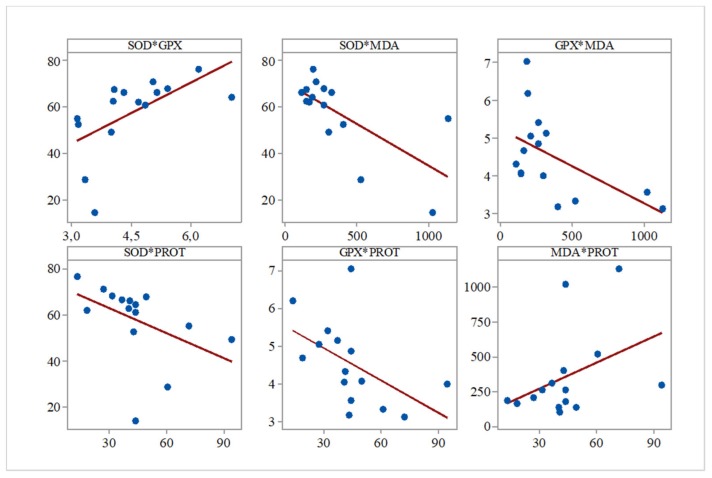
Correlation analysis of biochemical parameters (Pearson’s correlation, *n* = 15). Data expressed as: Superoxide dismutase (SOD) inhibition rate (%), U glutathione peroxidase (GPx)/mL, mmol malondialdehyde (MDA)/mL, mg proteins/mL.

Furthermore, while analyzing the Pearson correlations between the evaluated biochemical parameters for all the groups, we observed several significant correlations between SOD and GPx (r = 0.596, *p* = 0.019), SOD and PROT (r = −0.683, *p* = 0.005), GPx and PROT (r = −0.517, *p* = 0.048), and SOD and MDA (r = −0.541, *p* = 0.037) (Figure 1).

**Figure 2:**
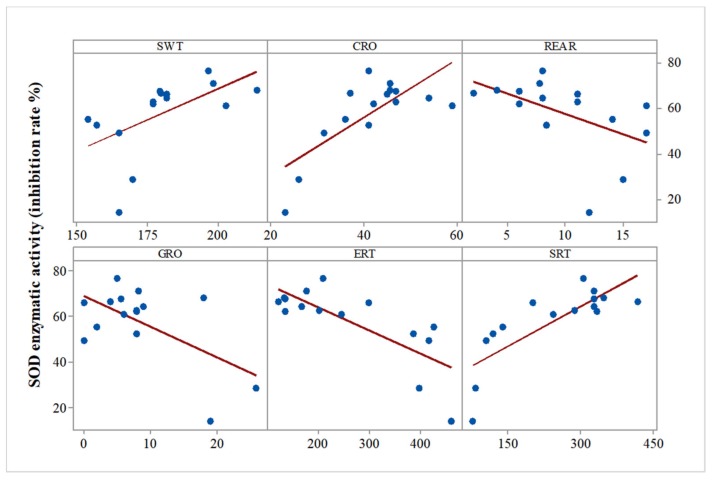
Correlation analysis of SOD enzyme activity versus the results of the behavioral tests (Pearson’s correlation, *n* = 15, SWT = swimming time, CRO = general mobility index, REAR = rearing, ERT = empty room time, GRO = grooming time, SRT = stranger room time). Data expressed as: time (s) for SWT, GRO, ERT, SRT, and events number for CRO, REAR.

Regarding the all-group Pearson correlations between biochemical parameters and previously described behavioral parameters [11], we found significant correlations between SOD activity and swimming time (SWT) (r = 0.567, *p* = 0.02), SOD activity and social preference (r = 0.788, *p* = 0.001), SOD activity and grooming time (r = −0.591, *p* = 0.02), SOD activity and general mobility (CRO) (r = 0.744, *p* = 0.001), SOD activity and rearing (r = −0.498, *p* = 0.05), and SOD activity and social isolation (ERT) (r = −0.767, *p* = 0.001) (Figure 2).

**Figure 3:**
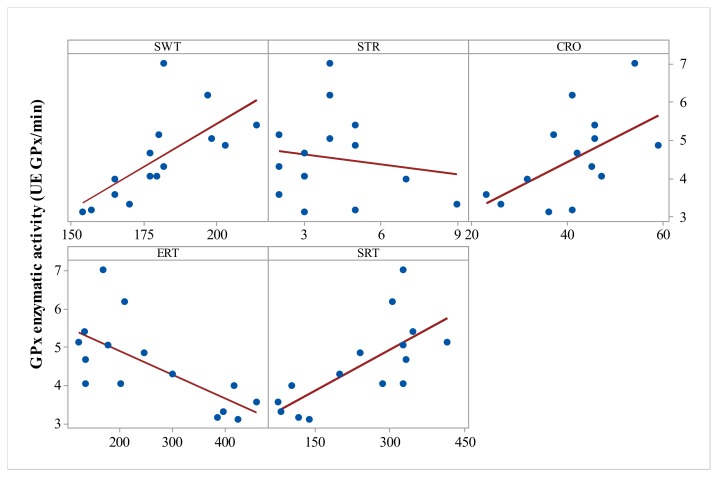
Correlation analysis of GPx enzyme activity and behavioral parameters (Pearson’s correlation, *n* = 15, SWT = swimming time, STR = stretching, CRO = crossings, ERT = empty room time, SRT = stranger room time). Data expressed as: time (s) for SWT, ERT, SRT, and events number for STR, CRO.

Significant all-group Pearson correlations were also observed between GPx activity and SWT (r = 0.685, *p* = 0.005), GPx activity and stretching behavior (r = −0.5, *p* = 0.05), GPx and CRO (r = 0.553, *p* = 0.033), and GPx activity and SRT (r = 0.715, *p* = 0.003) (Figure 3).

**Figure 4:**
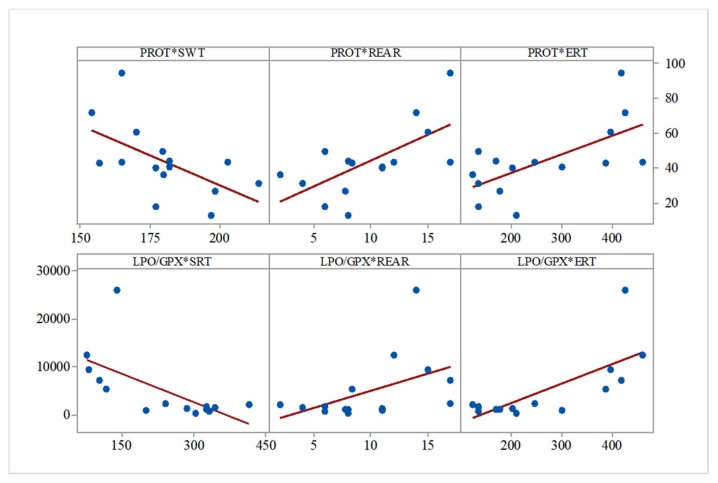
Correlation analysis of other biochemical parameters versus behavioral parameters (Pearson’s correlation, *n* = 15, SWT = swimming time, REAR = rearing, ERT = empty room time, SRT = stranger room time). Data expressed as: mg prot/mL, time (s) for SWT, ERT, and events number for STR, REAR.

In a similar manner, lipid peroxidation intensity correlated with swimming time (control and IBS: r = −0.565, *p* = 0.02), and total soluble proteins levels correlated with SWT (r = −0.578, *p* = 0.02), rearing behavior (r = 0.661, *p* = 0.007), and ERT (r = 0.647, *p* = 0.009) (Figure 4).

The peroxidation/hydrolysis ratio correlated with social preference (r = −0.642, *p* = 0.01), rearing behavior (r = 0.476, *p* = 0.07), and ERT (r = 0.734, *p* = 0.002) (Figure 4).

## 4. Discussion

This study aimed to describe brain oxidative stress parameters in a contention-stress IBS rat model. Relative to temporal brain extracts from control animals, those from rats subjected to contention exhibited decreases in the activity of antioxidant enzymes (SOD and GPx) and increases in lipid peroxidation and total soluble proteins levels. Moreover, treating the contention-stressed rats with a typical antidepressant agent (nortriptyline) induced significant improvements in brain oxidative stress.

IBS is reportedly associated with changes in oxidative stress in colonic cells, intestine homogenate, and blood sera [12,13,14,15]. As this study also observed changes in the oxidative stress status in central nervous system tissues, such findings provide evidence for the validity of the IBS animal model based on the induction of contention stress.

The negative correlation between the levels of antioxidant enzymes and lipid peroxidation markers suggests that the decrease in SOD activity may account for the intensity of lipid peroxidation. However, while a significant correlation between SOD and GPx activities was observed, no significant correlation was found between GPx activity and lipid peroxidation intensity. These findings indicate an increase that the activity of another antioxidant enzyme (catalase) may be attributable to the compensatory increase of total soluble protein levels, which positively correlates with lipid peroxidation.

Moreover, as changes can also be observed in behavior—specifically, the exacerbation of depression and anxiety spectrum symptomatology—we tested the administration of nortriptyline to verify the following hypothesis: changes in oxidative stress status correlate with behavioral patterns in the contention-stressed animals. We found complex correlations between depressive and anxious behavioral patterns and oxidative markers (antioxidant enzymes activity such as SOD and GPX), suggesting the possibility of modulating oxidative status components and depressive and anxious neurological pathways. Our results thus show that the contention-stress IBS animal model features value additional to that previously described by Mozaffari et al. [18].

Changes in brain tissue oxidative stress status were similar to those observed in systemic and local biochemical markers in other IBS models and patients. Regarding the oxidative stress patterns in IBS patients, while Ramírez-García et al. and Choghakhori et al. found low antioxidant activity, Oran et al. and Mete et al. found intense lipid peroxidation processes and high inflammatory cytokines activity in blood sera [25,26,27,28]. Furthermore, a recent study indicates several of these oxidative markers, as well as some inflammatory parameters, to be IBS biomarkers [29].

Similarly, for IBS animal models, research initiatives confirm the presence of increased inflammatory and lipid peroxidation markers in rats subjected to contention-stress. Asadi-Shahmirzadi et al. describe the beneficial effects of *Aloe vera* and *Matricaria recutita* mixture in a similar 5-days contention-stress rat model mentioning the changes in some oxidative stress markers in colonic cells; they also reported reduced antioxidant capacity, which was corrected by the use of antioxidant and spasmolytic agents [12]. Moreover, Jafar Zamani et al. observed that sildenafil, a smooth muscle relaxing agent, increased total antioxidant capacity in an IBS model by using wrap-restraint method [13]. Increased intestine homogenate lipid peroxidation was reported by Colares et al. in an acetic acid-induced IBS rat model while testing potential benefits of lecithin treatment in IBS [14]. A study performed by Zhang et al. on the relevance of melatonin administration to a noise-stress gastrointestinal distress rat model demonstrated increased oxidative stress followed by increased lipid peroxidation in blood sera [15]. Similar results were obtained in the present study: our data showed low antioxidant activity and increased lipid peroxidation in temporal lobe extracts. Thus, the fact that we obtained oxidative status changes in the brain of an IBS animal model suggests that a complex neurological component plays an important role in IBS pathological mechanisms. Moreover, our findings regarding the nortriptyline potential to reverse brain oxidative stress which occurred in this model lead to a possible determinant implication of oxidative imbalance in IBS development mechanisms.

However, some limitations of our study must be mentioned and discussed. In our study, we chose contention-stress animal model to evaluate the oxidative status changes occurring in the brain and the effect of an antidepressant agent based on previous experiences and studies. Despite that this animal model offers valid modulation of behavioral and biochemical features of IBS, the designing method used homotypic stress factor administration. Thus, in chronic administration conditions, it could lead to behavioral progressive habituation and chronic biochemical response. Therefore, further research efforts should be considered to avoid these shortcomings by designing IBS animal models based on multiple stress factors administration in the way they daily occur. Moreover, further interests regarding this IBS animal model could address the study of the abovementioned molecules and plant extracts in order to grant therapeutical avenues in IBS management.

## 5. Conclusions

Our results show increased brain oxidative stress in contention-stress rat model. Moreover, we showed that the biochemical ratios that are used for evaluating the effectiveness of an antioxidant system on oxidative stress can be successfully described in this model, which could suggest that this contention-stress model may be successfully used in complementary and alternative medicine studies. Furthermore, the correlations between the behavioral patterns and biochemical oxidative stress features could suggest that this may be a complex model, which can successfully mimic IBS symptomatology, further providing evidence of a strong connection between the digestive system, enteric nervous system, and the central nervous system.

## Figures and Tables

**Table 1:** Biochemical oxidative stress parameters and ratios.

Biochemical Marker	Control Group (*n* = 5)	IBS Group (*n* = 5)	IBS + Nortriptyline Group (*n* = 5)	*p*-Value *
SOD (inhibition rate %)	64,604 ± 1037	40 ± 7967 ^a^	68,702 ± 2562 ^b^	0.0026
GPx (U/mL tissue extract)	4825 ± 0.563	3431 ± 0.159	5331 ± 0.234 ^c^	0.0084
MDA (mmol/mL tissue extract)	147,559 ± 12,983	674,993 ± 49,422 ^d^	249,512 ± 22,122 ^e,f^	0.0058
Total soluble proteins (mg proteins/mL tissue extract)	38,649 ± 5,342	62,846 ± 9642	30,340 ± 5,167 ^g^	0.018
SOD/GPx specific activity ratio	13,986 ± 1321	11,860 ± 2542	12,912 ± 0.314	0.67
MDA level/GPx specific activity ratio	1,189,768 ± 18,481	12,154,694 ± 1,542,55 ^h^	1,540,295 ± 367,177 ^i^	0.005

* One-way ANOVA analysis; Bonferroni corrected *t*-test: ^a^—IBS group versus Control group, *p* = 0.015; ^b^–IBS + Nor group versus IBS group, *p* = 0.008; ^c^-IBS + Nor group versus IBS group, *p* = 0.0001; ^d^-IBS group versus Control group, *p* = 0.014; ^e^-IBS + Nor group versus IBS group, *p* = 0.008; c-IBS + Nor group versus IBS group, *p* = 0.036; ^f^-IBS + Nor group versus Control group, *p* = 0.004; ^g^-IBS + Nor group versus IBS group, *p* = 0.017; ^h^-IBS group versus Control group, *p* = 0.017; ^i^–IBS + Nor group versus IBS group, *p* = 0.02.
